# An In-Plane Single-Photon Emitter Combining a Triangular Split-Ring Micro-Optical Resonator and a Colloidal Quantum Dot

**DOI:** 10.3390/nano15050335

**Published:** 2025-02-21

**Authors:** Kohki Mukai, Kyosuke Uchiyama, Kohei Iwata, Issei Pribyl

**Affiliations:** Graduate School of Engineering Science, Yokohama National University, Yokohama 240-8501, Japan

**Keywords:** single-photon emitter, colloidal quantum dot, micro-optical resonator, metamaterial, FIB lithography

## Abstract

We propose a simple and innovative configuration consisting of a quantum dot and micro-optical resonator that emits single photons with good directionality in a plane parallel to the substrate. In this device, a single quantum dot is placed as a light source between the slits of a triangular split-ring micro-optical resonator (SRR) supported in an optical polymer film with an air-bridge structure. Although most of the previous single photon emitters in solid-state devices emitted photons upward from the substrate, operation simulations confirmed that this configuration realizes lateral light emission in narrow regions above, below, left, and right in the optical polymer film, despite the absence of a light confinement structure such as an optical waveguide. This device can be fabricated using silica-coated colloidal quantum dots, focused ion beam (FIB) lithography, and wet etching using an oxide layer on a silicon substrate as a sacrificial layer. The device has a large tolerance to the variation in the position of the SRR in the optical polymer film and the height of the air-bridge. We confirmed that Pt-SRRs can be formed on the optical polymer film using FIB lithography. This simple lateral photon emitter is suitable for coupling with optical fibers and for fabricating planar optical quantum solid-state circuits, and is useful for the development of quantum information processing technology.

## 1. Introduction

Single photon emitters are key devices in quantum information processing technology that use photon states as qbits. Various types of single photon emitters have been proposed and fabricated to date, and all of them basically combine nanocrystals (such as quantum dots (QDs)) as light-emitting sources with optical resonant structures to control their properties. Representative optical resonant structures include photonic crystals [1,2,3] and metamaterials [4,5,6,7]. It has been reported that light-emitting properties can be strongly controlled by these optical resonant structures with high q values [8,9]. However, since photonic crystals and metamaterials are composed of repeated patterns of microstructures, the size of the entire device must be at least on the order of tens of microns. Therefore, for example, if multiple single photon emitters are combined to form a solid-state integrated circuit, a macroscopic structure must be manufactured with extremely high precision, which is not realistic. On the other hand, there are many reports of simple single photon sources that combine QDs and nanoantennas [10,11,12,13,14,15,16,17]. In most of these reports, colloidal QDs (CQDs) such as PbS QDs and CdS QDs were randomly scattered on the surface of a substrate on which multiple nanoantennas were formed, and single photon emission due to resonance in the area where the nanoantennas and CQDs happened to be in close proximity was observed. However, the manufacturing process does not allow the position of the light source to be predetermined, and therefore the device’s characteristics cannot be engineered. Such devices are clearly not fit for widespread use. Semiconductor epitaxy techniques are effective for fabricating reproducible devices [18], but epitaxial QDs have an anisotropic structure that is not suitable for emitting entangled photon pairs [19]. A method has been proposed to capture and control the position of CQDs using optical tweezers [20], but this requires constant irradiation of laser light while the device is in use and is not suitable for integration. Although a structure in which a single CQD is bound to the surface of an optical nanofiber has been proposed [21], its applications are limited by the use of optical fibers.

We have previously proposed a variety of single-photon emitters that combine position-controlled silica-coated QDs with a single unit of metamaterials that exhibit localized surface plasmon resonance. Silica-coated QDs are silica spheres grown in reverse micelles with CQDs as cores [22,23,24,25,26,27]. Silica coating on CQDs does not change the energy state of the CQDs much, but it increases the effective size of the CQDs, making them much easier to handle and allowing position control. The diameter of silica-coated QDs ranges from approximately 10 nm to 150 nm. Nanoholes of this size can be formed, for example using scanning probe microscope (SPM) lithography, and silica-coated QDs can be trapped in them. When combined with a single unit of metamaterials, the emission from the CQDs is controlled by the localized surface plasmons in the metamaterial unit. We have demonstrated that optical properties such as the upward directionality and polarization of emitted photons can be controlled by a single metamaterial unit, such as a quadrilateral split-ring resonator (SRR) [28], a double circular SRR [29], and an elliptical SRR [30]. On the other hand, single photon sources that emit photons in the in-plane direction parallel to the substrate are suitable for the realization of quantum planar circuits. Also, light emission in the lateral direction can be connected to optical fibers similar to semiconductor lasers and is valuable for applications. We have previously proposed a lateral single photon emitter that combines a single QD and a micromachined optical resonator with an optical waveguide as a cantilever [31]. The timing of the photon emission of this device can be controlled by micromachining, but a complex fabrication process is required. In this study, we propose a relatively easy-to-fabricate single photon emitter with lateral directionality by combining a silica-coated QD and a triangular SRR metamaterial unit.

## 2. Device Structure and Basic Performance

Various shapes of SRR structures for lateral light emission were first investigated via optical simulation using the finite-difference time-domain method. The simulation software used was CST Studio Suite (Dassault Systems Simulia Corp., Paris, France). When semiconductor lithography is used as a manufacturing method, planar shapes are easy to fabricate, so the SRR structures investigated were limited to two-dimensional ones. The light source was a dipole located in the horizontal plane of the substrate. In this case, light can be emitted in both horizontal and vertical directions relative to the substrate, and the light emission characteristics in three-dimensional directions can be evaluated. The wavelength of the light source was assumed to be 1300 nm (230.6 THz), which is the wavelength in the optical communication band. The resonant wavelength was confirmed by the wavelength dependence of the S parameters, and the dBi in the light emission direction was used as an index to evaluate the directivity.

After examining structures such as circular SRRs, triangular SRRs, square SRRs, and Euro-shaped resonators [32], it was found that the single triangular SRR was capable of emitting light with directivity in the lateral direction. In metamaterial units with double identical shapes, whether circular, triangular, or square, it was found that in order to resonate at a wavelength of about 1300 nm, the inner structure must be so small that it is difficult to manufacture. In the single square SRR and Euro-shaped resonators, it was difficult to suppress the emission in the vertically upward direction due to the in-plane symmetric structure, and the same was true when a light source was placed between the split slits. However, it was found that the single triangular SRR was capable of emitting light with good directivity in the lateral direction when a light source was placed between the slits. The light source placed between the slits generates electric field oscillations preferentially between the ends of the slits, and the surface plasmons induced by the electric field in the SRRs determine the radiation characteristics. Although there have been other reports of triangular SRRs [33,34], we believe that we are the first to use them for lateral light emission. The circular SRR was also able to emit light in the lateral direction with a similar technique, but the directivity of the triangular SRR was clearly superior. The linear structure of the triangular SRR also has the advantage of being relatively easy to fabricate.

When determining the basic shape of the SRR, we assumed that the surroundings were a vacuum, but we then considered how to actually place the SRR. We performed simulations on silicon, gold, silica glass, and polymethyl methacrylate (PMMA) substrates to confirm the directivity, but no matter what substrate we assumed, we were unable to obtain sufficient lateral directivity due to the vertical asymmetry of the structure. Therefore, we decided to create an air bridge structure in which the SRR is supported within PMMA, an optical polymer, and to create it by etching a sacrificial layer using a silicon substrate.

**Figure 1:**
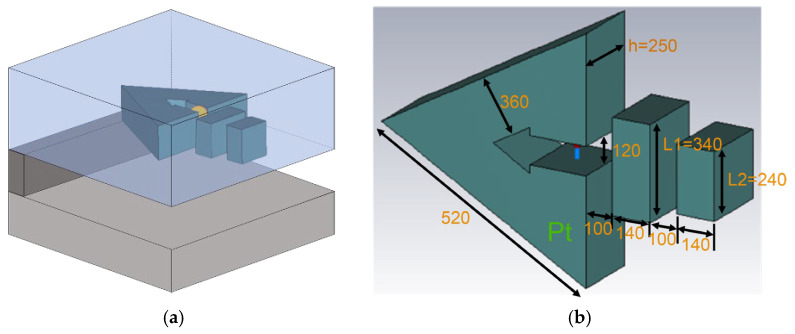
The proposed single photon emitter with a triangular SRR metamaterial unit arranged around a QD. (**a**) A bird’s-eye view of the device, and (**b**) the design dimensions (units in nanometers).

Figure 1 shows the single photon emitter in which QDs are placed inside the metamaterial unit of the triangular SRR that we propose in this study. A slit is made on one side of the triangular SRR, and the QDs are placed between the slits as a light source. The light emission characteristics in the lateral direction are improved by adding a nanoantenna near the slit of the triangular SRR. Figure 1a shows a bird’s-eye view of the device. The whole device is supported in a 1 µm thick PMMA film, forming an air bridge structure. Figure 1b shows the design dimensions of the triangular SRR and two nanoantennas placed in front of the slit. The material of the SRR is Pt, which can be fabricated in our focused ion beam (FIB) facility. This dimension can be fabricated via FIB lithography without much difficulty. The main SRR portion is an equilateral triangle with each side being 520 nm long. The width of the slit is 120 nm, which is wide enough to place the silica-coated QDs between them. The thickness of the SRR is 250 nm, and it is assumed that the light source is exactly in the center of the thickness of the SRR.

**Figure 2:**
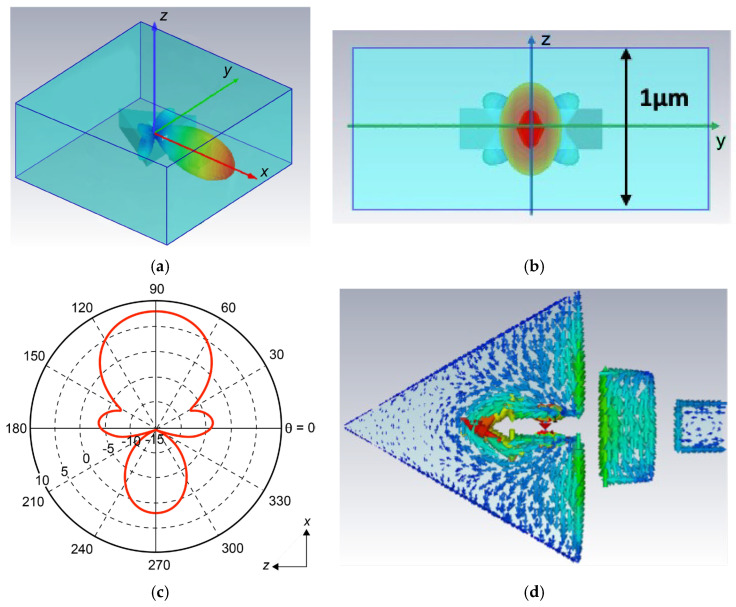
The simulation results of the light emission characteristics of the device. (**a**) The resonant 227.6 THz light emission, and (**b**) the view of the emitted light from the direction of emission, (**c**) the emitted light pattern showing directivity, and (**d**) the electric field inside the SRR.

Figure 2 shows the simulation results of the light emission characteristics of this device. Here, we assume that the air bridge is sufficiently high. Figure 2a is a bird’s-eye view of the resonant 227.6 THz light emission. Reflecting the vertical symmetry of the structure, it can be seen that the light is strongly emitted in the lateral direction. Figure 2b shows the light emission seen from the exit side in the x direction. It can be seen that the area from which the light is emitted is much narrower in the vertical direction than the thickness of the PMMA. It can also be seen that the light is confined in a very narrow area in the lateral direction even though there is no lateral light confinement structure such as an optical waveguide. Figure 2c shows the emission light pattern that shows the directivity of this device. θ = 0° is the substrate direction, 180° is vertically above the substrate, and 90° is the antenna direction. It can be clearly seen that the light is emitted from this device in the lateral direction in the substrate plane with a very high directivity of 8.36 dBi toward the antenna. The back lobe is extremely weak, and the side lobe is almost nonexistent. In the simulation shown here, the dipole vibration direction was set to the y direction, which was the case where the light source and SRR had the strongest resonance. When the dipole vibration direction was set to the x- or z-direction, the light emission was not necessarily strongest in the direction of the nanoantenna, and even in those cases, the maximum emission intensity was at least an order of magnitude lower than that shown here. Figure 2d shows the electric field in the x–y plane inside the SRR. It can be seen that strong electric field resonance occurs at two points: the slit where the light source is installed and the deep part of the ring, achieving high directivity.

**Figure 3:**
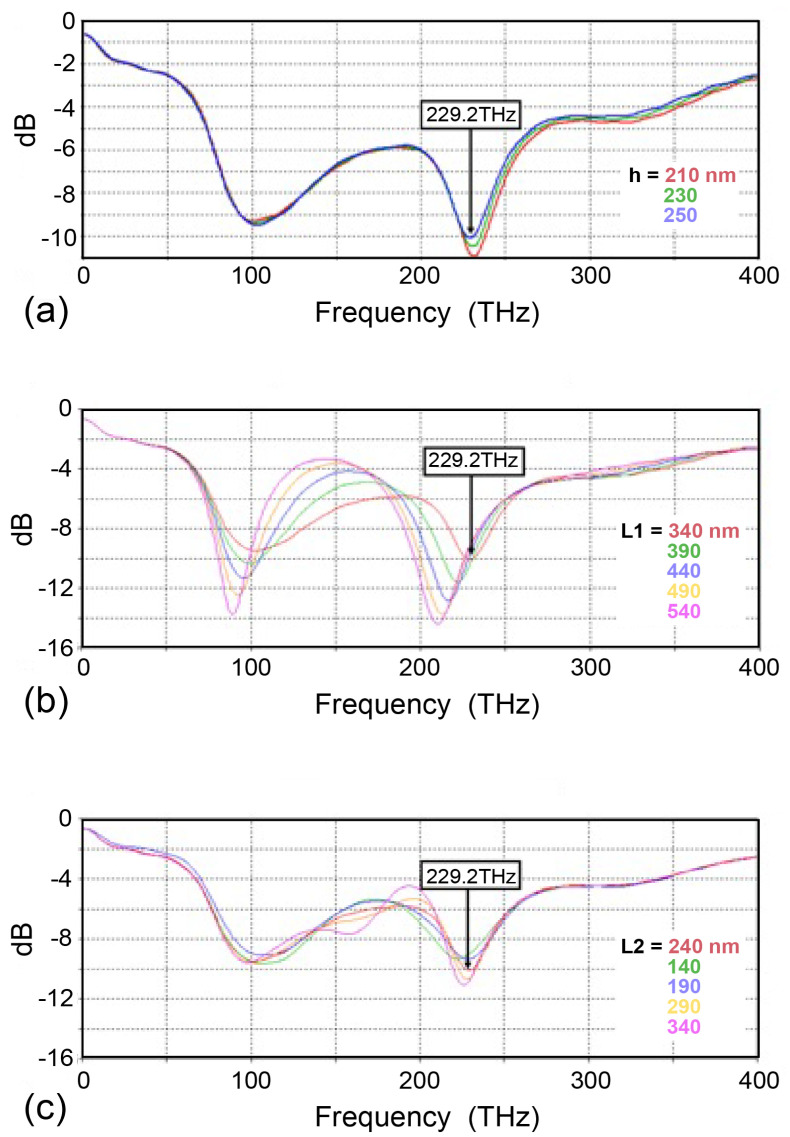
The effect of the deviation of the structure dimensions on the S11 parameter. (**a**) The SRR thickness, (**b**) the length of the nanoantenna close to the SRR, and (**c**) the length of the nanoantenna far from the SRR.

Figure 3 shows an example of the effect of the SRR dimensions on the resonance characteristics, which was considered when determining the optimal structure. Figure 3a shows the effect of the SRR thickness. Even if the thickness is reduced by about 20%, the resonance characteristics are hardly affected. The q value estimated from these spectral widths was about 5.8. In general, metamaterial systems using localized surface plasmon resonance are known to have low q values [35,36,37,38,39], and our SRR is no exception. Figure 3b shows the effect of the length of the nanoantenna closer to the SRR. It is sensitive when the change is relatively small, and when the length is increased by about 20% (340 nm to 390 nm), the resonance wavelength shifts to the lower frequency side by about 10 THz. However, even if the length is increased further by about 60% (340 nm to 540 nm), the shift of the resonance wavelength is limited to about 20 THz. Figure 3c shows the effect of the nanoantenna length on the side farther from the SRR. When the length is changed in the range from −30% to +40% (240 nm to 340 nm), both the resonance wavelength and the strength of the resonance change little by little. In other words, it is suggested that the length of the nanoantenna farther from the SRR is good for fine tuning the resonance wavelength. 

## 3. Fabrication of SRR and Tolerance of Manufacturing Errors

The designed device can be fabricated by the following process. First, a thermal oxide layer is generated on the surface of a Si substrate by heat-treating it in an oxygen atmosphere in a furnace. For example, a sacrificial layer of about 1 μm is obtained by holding it at 950 °C for 60 h [40]. Next, PMMA with a thickness of about 375 nm is spin-coated on the thermal oxide layer. This is a base layer that takes into consideration that the SRR with the designed thickness will be located in the center of the PMMA film with a thickness of 1 μm in the end. Next, a Pt SRR with a height of 250 nm is fabricated on the PMMA using FIB lithography. Then, a 250 nm thick PMMA layer is spin-coated so that it just fills the SRR. At this stage, a light source, which is a silica-coated QD, is installed in the following procedure. A nanohole that matches the size of the silica-coated QD is created in the slit part of the SRR. It would be efficient to use the etching function of the FIB. Next, a solution of silica-coated QDs is dropped and the unnecessary silica-coated QDs are removed by wiping with an industrial cotton swab or the like. The nanohole diameter is adjusted to approximately match the diameter of the silica-coated QD so that one silica-coated QD can fit into one nanohole. For further details, see [27]. PMMA is then spin-coated to a total thickness of 1 μm. The resulting multilayer film is cut using FIB etching near the light-emitting end face of the SRR, the Si substrate is cleaved, and the silicon oxide sacrificial layer is removed via wet etching to form an air bridge structure, completing the device. 

**Figure 4:**
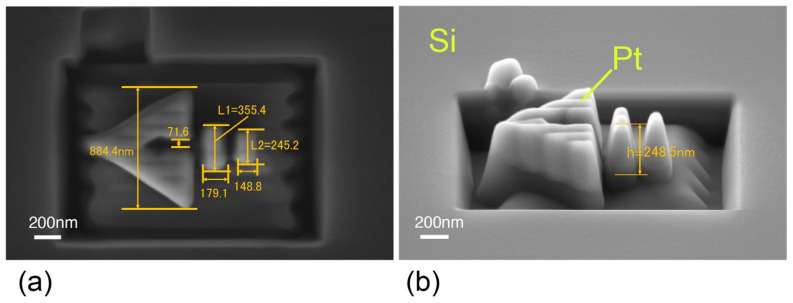
The prototype of SRR made of Pt on a PMMA layer. (**a**) Device dimensions from the top, and (**b**) device dimensions from the side.

Figure 4 shows an example of a prototype SRR made of Pt on a PMMA layer. An FIB system (Crossbeam 550, Carl Zeiss, Oberkochen, Germany) was used to fabricate the SRR. The PMMA layer is non-conductive, and the silicon oxide sacrificial layer further reduces the conductivity with the substrate, so if no countermeasures are taken, the PMMA layer will charge up, making the FIB processing difficult. Therefore, in this prototype, a countermeasure against charge-up was taken by covering the area near the SRR formation site with conductive tape. This may not be the case when the PMMA layer is extremely thick, but the charge-up was sufficiently eliminated with a PMMA layer of less than 500 nm as in this study. After taking this countermeasure, a Pt square protrusion was first deposited on the PMMA, and the SRR shape was cut out via ion etching. The deviation from the design value of the completed SRR was less than 5% in the plane and less than 1% in height. In Figure 4b, the boundary between the Pt SSR and the underlying PMMA can be confirmed by the difference in color. As is clear from these figures, in the processing in the height direction, over-etching progresses to the PMMA layer, resulting in the formation of a rectangular recessed shape around the SRR. This recessed part is expected to hold the CQDs in the process of trapping the CQDs in the nanoholes, and must be eliminated. Although texture and cone-shaped protrusions are seen, we believe that these can also be improved by adjusting the FIB manufacturing process. It can also be seen that there is some problem with the perpendicularity of the FIB etching. If the perpendicularity cannot be sufficiently improved, it will be necessary to redo the design simulation taking into account the shape in the height direction. These technical problems in FIB processing are not essential, and it is believed that they can be resolved to some extent by optimizing the processing conditions. One way to deal with over-etching is to fabricate the SRR via deposition alone.

One of the difficulties in fabricating the device is the placement of the SRR at the center of the multi-layer PMMA layer. We therefore simulated the directivity and S11 parameters while moving the SRR in the thickness direction by 50 nm from the center to the upper limit of the PMMA layer. Although not shown here, the results showed that there was little difference in the emission pattern and the spectrum of the S11 parameters depending on the position in this range. As a result, it was found that the vertical position of the SRR can be anywhere within the 1 μm thick PMMA layer. 

**Figure 5:**
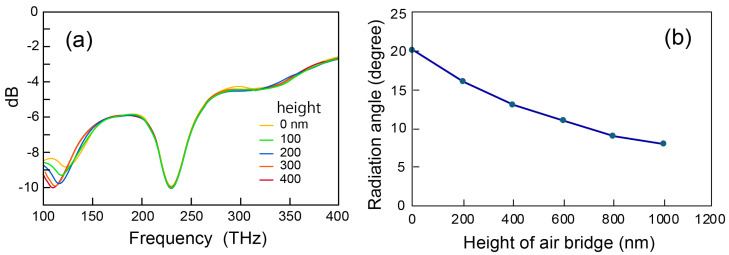
The effect of increasing the height of the air bridge. (**a**) The S11 parameter spectrum, (**b**) the light emission angle.

Although the air bridge structure ensures the structural symmetry of the device, it is not possible to fabricate an extremely high air bridge. Therefore, the effect of the air bridge height was evaluated via simulation. Figure 5a shows the change in the S11 parameter when the air bridge height was increased. As shown here, the shape of the spectrum changes slightly, but the position of the resonant frequency peak hardly changes. Figure 5b shows the dependence of the light emission angle on the height of the air bridge. The horizontal direction is 0 degrees, and the upward direction is positive. When there is no air bridge structure, light is emitted upward at an angle of 20 degrees, and as the thickness of the air layer in the air bridge structure increases, the light emission angle becomes smaller, reaching about 8 degrees at a thickness of 1 µm. The effect of increasing the air bridge height beyond this could not be evaluated because the increase in the number of meshes for spatial division in the simulation exceeded the memory capacity of our computer. However, the decrease in the light emission angle becomes smaller beyond this. By extrapolating this data, it was estimated that the emission angle becomes 1 degree or less when the air bridge height is about 11 µm. On the other hand, the realistic thickness of the sacrificial layer formed by thermal oxidation is about several µm. Although it is difficult to obtain perfect lateral emission via the method using thermal oxidation, it is thought that even with an air bridge height of several µm, it is possible to keep it within the NA of the optical waveguide or optical fiber placed in the lateral direction.

We have shown that the SRRs required for the proposed single-photon emitter can be fabricated with sufficient precision using FIB, and the required air bridge height was also adequate. The remaining challenge for completing the device is to provide a good single-photon light source. This device assumes that the light source is a silica-coated QD. To trap QDs using nanoholes, it is desirable to use silica-coated QDs as large as possible, but it is known that the thicker the silica coating, the lower the light emission efficiency [27,41,42]. The slit width of the SRR used in this device was 120 nm, and the size of the silica-coated QD required is less than that. However, at present, the emission intensity begins to weaken when the particle size is 50 nm or more, and the emission becomes extremely weak when the particle size exceeds about 100 nm. For stable operation of the device, the single QD installed must always emit photons, so the development of a large-sized good light source is required. We are currently working on developing such silica-coated QDs, and if successful, we plan to fabricate prototype devices and evaluate their emission directionality and optical resonance properties. 

## 4. Conclusions

We found that a device that emits photons with good directionality parallel to the substrate can be fabricated by placing CQDs in the slits of triangular SRRs and supporting them on an optical polymer film with an air-bridge structure. The light emission of this device occurs in a small area, despite the absence of a light confinement structure such as an optical waveguide. This device can be realized by using FIB lithography and wet etching using an oxide layer on a silicon substrate as a sacrificial layer. It was also confirmed that there is a large tolerance for the position of the SRRs in the PMMA layer and the height of the air-bridge. This simple device, which emits photons parallel to the substrate, is advantageous for coupling single photons into optical fibers and for constructing solid-state photonic quantum circuits. We believe it will contribute to the development of quantum information processing and related fields.

## Data Availability

Data will be made available on request.
